# The Effects of a Novel Sodium Bicarbonate Ingestion System on Repeated 4 km Cycling Time Trial Performance in Well-Trained Male Cyclists

**DOI:** 10.1007/s40279-024-02083-4

**Published:** 2024-08-09

**Authors:** Lewis A. Gough, S. Andy Sparks

**Affiliations:** 1 Human Performance and Health Research Group, Birmingham City University, Birmingham, B15 3TN UK; 2 Maurten AB, Gothenburg, Sweden; 3 Research Institute for Sport and Exercise Sciences, Liverpool John Moores University, Liverpool, UK

## Abstract

**Background:**

A novel sodium bicarbonate (SB) product has come to market named the “Bicarb System” (M-SB; Maurten AB, Gothenburg, Sweden). It claims to minimise gastrointestinal (GI) discomfort whilst still improving exercise performance.

**Aim:**

To investigate the effects of M-SB ingestion on repeated 4 km cycling time trials (TT1 and TT2) in well-trained male cyclists.

**Methods:**

The study recruited ten well-trained cyclists (maximal oxygen uptake ($\dot{V}{\text{O}}_{2\max }$): 67 ± 4 ml kg^−1^ min^−1^ BM; peak power output (PPO) at $\dot{V}{\text{O}}_{2\max }$: 423 ± 21 W) to take part in this randomised, crossover and double-blinded study. Following one visit to determine $\dot{V}{\text{O}}_{2\max }$, participants completed a second visit to identify individual time to peak blood bicarbonate (HCO_3_^−^) (ITTP) in a rested state. Visit three was a familiarisation trial mimicking the experimental procedures. Visits four to seven consisted of completing 2 × 4 km cycling TTs separated by 45 min passive recovery, following one of either: 0.3 g kg^−1^ BM M-SB, 0.21 g kg^−1^ BM sodium chloride (placebo; PLA) in vegetarian capsules (size 00), or a control trial (CON). Supplements (M-SB or placebo) were ingested pre-exercise at their respective ITTP.

**Results:**

Performance in TT1 was faster in the M-SB condition compared with TT1 in CON (− 5.1 s; *p* = 0.004) and PLA (− 3.5 s; *p* < 0.001). In TT2, performance was also significantly faster in the M-SB condition compared with CON (− 4.4 s; *p* = 0.018) or PLA (− 4.1 s; *p* = 0.002). Total aggregated GI symptoms were generally low and not significantly different between PLA and the M-SB conditions for a range of symptoms.

**Conclusions:**

The ingestion of M-SB improves repeated 4 km cycling TT performance and the recovery of acid–base balance between bouts, whilst causing minimal GI discomfort.

## Key Points

**Table:** 

The ingestion of the novel “Bicarb System” (M-SB) improved repeated 4 km cycling time trial (TT) performance compared with placebo. Athletes should therefore consider using M-SB to improve performance.
Limited gastrointestinal (GI) discomfort was reported following M-SB ingestion making this an attractive SB ingestion method, as ergogenic effects can be achieved with limited side effects.
The ingestion of M-SB improved acid–base balance recovery during a 45 min passive rest and subsequent exercise performance. These findings may be applicable to training and/or competition where only a short recovery time frame is available (e.g. heat and final events).

## Introduction

Sodium bicarbonate (SB) is a popular ergogenic aid predominantly associated with improving high-intensity exercise performance [1]. The mechanism of action is related to the regulation of acid–base balance under conditions of high hydrogen ion (H^+^) production. A dose of SB in the range of 0.2–0.4 g^**.**^kg^−1^ body mass (BM) can increase the extracellular blood bicarbonate (HCO_3_^−^) concentration by approximately 5 mmol L^−1^. This leads to an increase in buffering capacity and therefore the rate of efflux of H^+^ from intracellular to extracellular compartments [2]. These changes in blood alkalosis could be beneficial to exercise performance (although debated; see [3]), as high H^+^ accumulation is generally considered to contribute to fatigue by impeding calcium (Ca^2+^) release from the sarcoplasmic reticulum, increasing the rate of inorganic phosphate accumulation and altering the strong ion difference (SID) [4, 5]. The effects related to the movement of other ions could also explain the improvements in performance, as the collective changes in ions [i.e. SID, including sodium (Na^+^), Ca^2+^, chloride (Cl^−^), and potassium (K^+^)] could lead to improved muscle action potential by increasing cell membrane potential and excitability [6]. Indeed, Gough et al. [7] reported following ingestion of 0.3 g kg^−1^ BM SB significantly lower extracellular K^+^ and Cl^−^ and an increased Na^+^ compared with placebo. These combined changes would collectively increase the SID and potentially benefit muscle contraction. Other researchers have also corroborated these findings when investigating movements of multiple or singular ions (e.g. K^+^; [8–10]). On the basis of this evidence, more research is required investigating both the H^+^ and HCO_3_^−^ mechanisms of SB ingestion alongside movements in singular ions.

Whilst there is considerable evidence that an ergogenic effect can be observed with ingestion of 0.3 g kg^−1^ BM SB in cycling time trials (TTs) [11], one limiting factor to SB ingestion is the gastrointestinal (GI) discomfort that can follow approximately 60–90 min after ingestion. Symptoms such as stomach bloating and pain, diarrhoea and nausea are common [12]. These side effects could disrupt the potential for an ergogenic effect [13, 14] or deter athletes from SB ingestion entirely. Whilst attempts have been made to mitigate the GI discomfort following ingestion, these have not always been successful. Specifically, Carr et al. [15] reported that ingestion of a carbohydrate (CHO) meal alongside ingestion of SB in capsules reduced GI discomfort compared with other ingestion methods (e.g. solution or capsules without a CHO meal). Nonetheless, GI discomfort was still present to a moderate level. A later series of experiments by Hilton and colleagues [16, 17] reported that ingestion of enterically coated SB reduced GI discomfort, although it was still present in some individuals to a moderate level, and in some cases, diarrhoea occurred several hours after the protocol had finished. On the basis of this evidence, further strategies are required to reduce the GI discomfort following SB ingestion, particularly as athletes have a fear of supplementation due to these potential adverse side effects.

Recently, a novel SB product named the “Maurten Bicarb System” (M-SB; Maurten AB, Gothenburg, Sweden) has been developed and is now commercially available. This system comprises a minitablet design in which each one is 3 mm in diameter and 1.5 mm in height. These minitablets are ingested alongside a CHO hydrogel gel that is suggested to reduce the interaction of SB with stomach acid and facilitate digestion. Importantly, recent data suggested M-SB can increase blood HCO_3_^−^ and almost eliminate GI discomfort compared with SB ingestion in vegetarian capsules [18]. Indeed, Gough and Sparks [18] reported that the increase in HCO_3_^−^ from baseline to individual time to peak (ITTP) was 8.2 ± 2.1 mmol L^−1^, which is considerably higher than the 5 mmol L^−1^ threshold purported to lead to ergogenic effects [15]. The increase in blood HCO_3_^−^ was also significantly greater following M-SB ingestion compared with an identical 0.3 g kg^−1^ BM dose of SB administered in vegetarian capsules. Perhaps more importantly, the ingestion of M-SB reduced aggregated GI discomfort significantly by 80 arbitrary units (AU) compared with vegetarian capsules (10 versus 90 AU) with M-SB almost eliminating GI discomfort in participants with a history of GI symptoms. This study did not, however, feature a trial to investigate the effects of M-SB ingestion on exercise performance.

A number of studies have shown that SB ingestion may improve repeated high-intensity exercise performance, and this could be via an improved recovery of acid–base balance between the two bouts of exercise [7, 9]. This has important practical implications for cyclists in the scenario that a limited time frame is available between bouts of exercise (such as heat and final scenarios in track cycling). Nonetheless, it is currently unknown whether M-SB ingestion can improve exercise performance and/or acid–base balance recovery. The purpose of this study therefore was to investigate the effects of M-SB ingestion on repeated 4 km cycling time trial performance in well-trained male cyclists.

## Methods

### Participants

Ten well-trained male cyclists volunteered for this study [age: 31 ± 8 years; mass: 76 ± 5 kg; maximal oxygen consumption ($\dot{V}{\text{O}}_{2\max }$): 67 ± 4 ml kg^−1^ min^−1^ BM; peak power output (PPO) at $\dot{V}{\text{O}}_{2\max }$: 423 ± 21 W)]. All cyclists met the criteria of ‘well trained’ as defined by De Pauw et al. [19] by meeting the threshold required for $\dot{V}{\text{O}}_{2\max }$ and PPO. All participants were part of a UK cycling club and trained > 3 times for ≥ 10 h/week [19]. The study received university ethics committee approval (Birmingham City University: #10651/sub2/R(B)/2022/May/HELS FAEC) and participants voluntarily provided written informed consent to take part. The study was performed in accordance with the ethical standards in the 1964 Declaration of Helsinki and later amendments.

### Experimental Overview

**Fig. 1:**
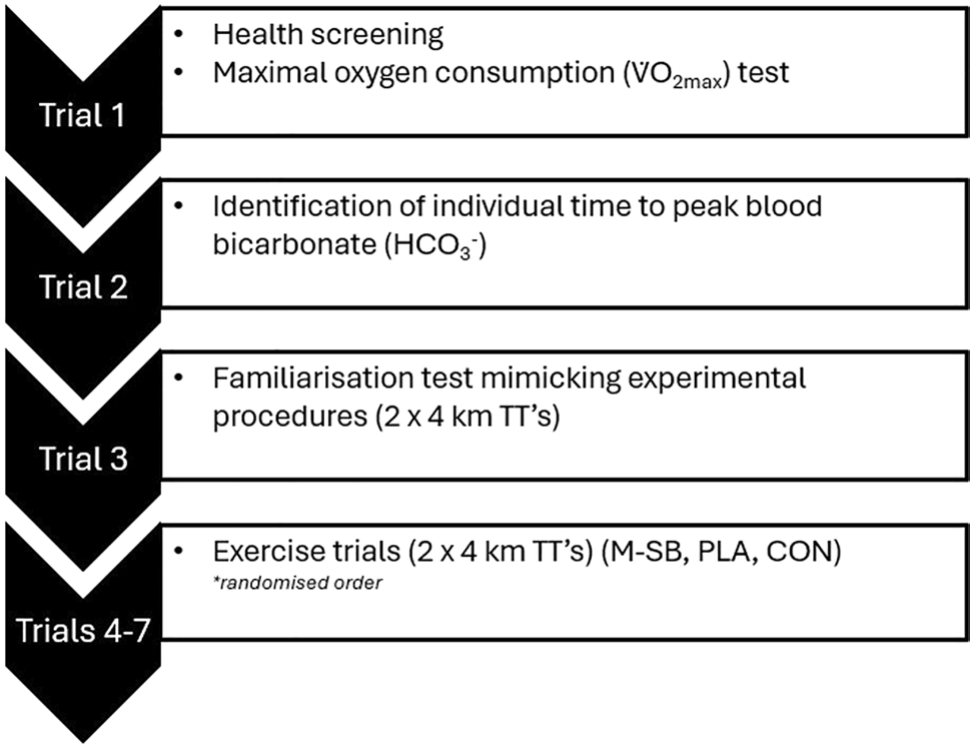
Schematic representation of the study design describing each trial procedures. *TT* time trial, *km* kilometre, *PLA* placebo, *CON* control

**Fig. 2:**
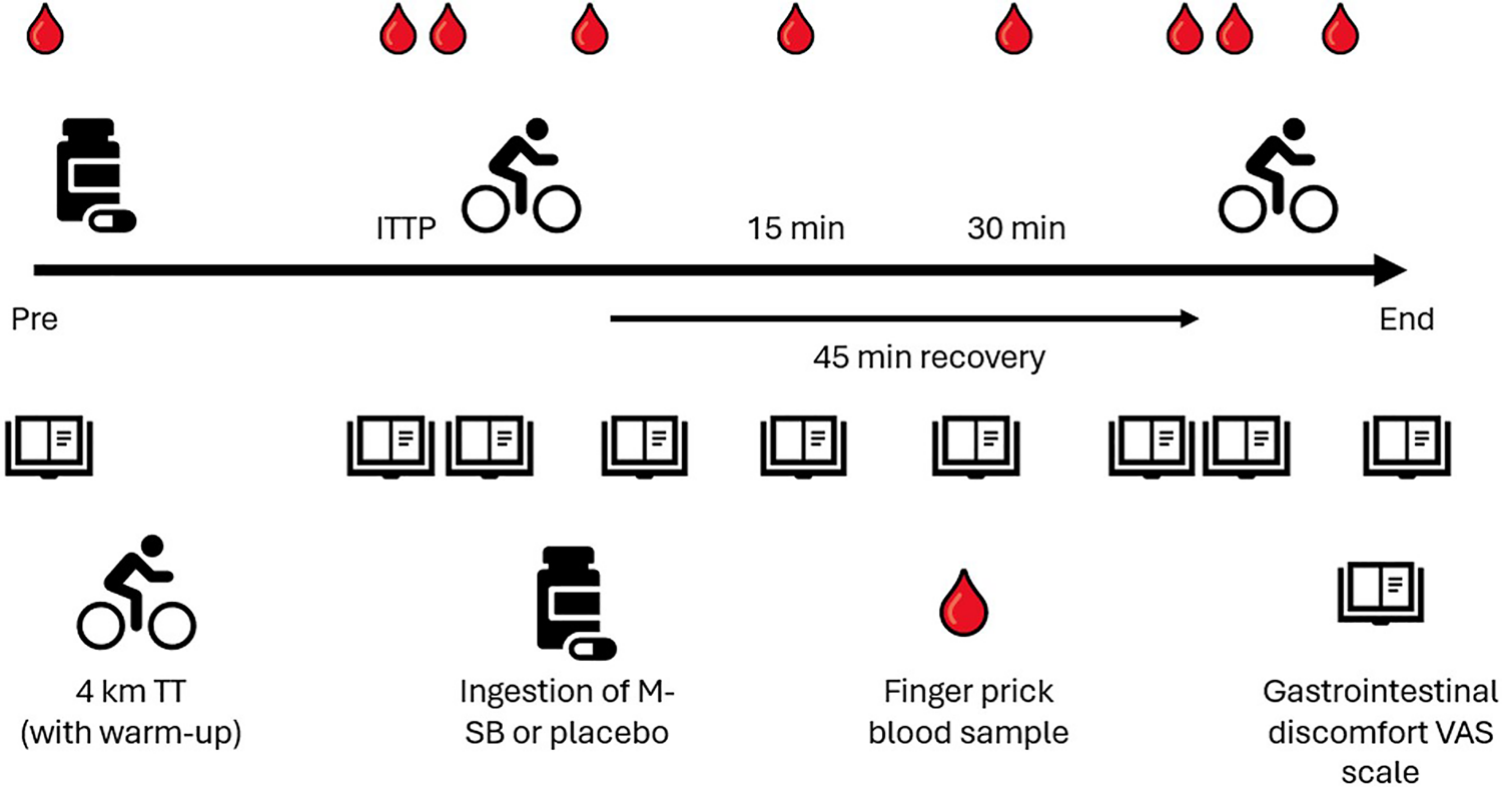
Schematic representation of the exercise trials. Fingertip blood samples were taken for pH, bicarbonate (HCO_3_^−^), sodium (Na^+^), potassium (K^+^), calcium (Ca^2+^), chloride (Cl^−^), and lactate; no supplement was ingested for the control trial. *ITTP* individual time to peak, *VAS* visual analogue scale

This study employed a randomised, crossover, placebo-controlled and double blinded design, and consisted of seven visits (Fig. 1). In the first visit, participants completed pre-trial health screening (PAR-Q) and a $\dot{V}{\text{O}}_{2\max }$ test. On the second visit, an individual time to peak blood bicarbonate (ITTP) test was conducted following ingestion of 0.3 g kg^−1^ BM SB (Maurten AB, Gothenburg, Sweden; M-SB). The third visit was a familiarisation trial consisting of completing 2 × 4 km cycling time trials (TT) on a cycle ergometer (Wattbike, Nottingham, UK), separated by a 45 min passive recovery period. Using the previously identified ITTP, three separate trials were then conducted where participants completed the two TTs with the recovery period between them, following the ingestion of M-SB, placebo (PLA) or a control (CON) in a block randomised order (Fig. 2).

Participants refrained from ingesting caffeine, alcohol and from performing exhaustive or prolonged exercise for 24 h prior to each trial. All trials were conducted at a similar time of day (± 1 h) to control for circadian variations. Nutritional intake was recorded via 24 h dietary recall and then replicated for each trial, using the ‘snap-n-send’ and written methods [20]. Participants were encouraged to ingest a meal consisting of 2 g kg^−1^ BM CHO approximately 2 h prior to all trials to replicate the typical preparation of highly trained cyclists prior to exercise. Participants achieved an intake of 1.7 ± 0.2 g kg^−1^ BM CHO, along with 19 ± 6 g of protein and 12 ± 4 g of fat, totalling a pre-exercise meal energy intake of 702 ± 99 kcal.

### Experimental Procedures

#### Maximal Oxygen Consumption

To determine the training status of the participants, they first completed a graded exercise test for the determination of $\dot{V}{\text{O}}_{2\max }$. Following a warm-up, participants completed a graded exercise test that began at 75 W and increased by 25 W min^−1^ to volitional exhaustion. Participants performed the test with a consistent cadence (85–95 rev min^−1^) whilst their oxygen consumption ($\dot{V}{\text{O}}_{2}$), carbon dioxide ($\dot{V}{\text{CO}}_{2}$) and respiratory exchange ratio (RER) were measured continuously (breath-by-breath) using a gas analyser (Metalyser, Cortex, Germany). Following the test, determination of $\dot{V}{\text{O}}_{2\max }$ was defined as the highest plateau reached with two successive readings within 0.15 L min^−1^.

#### Identification of Individual Time to Peak Blood Bicarbonate

On a separate visit, participants ingested 0.3 g kg^−1^ BM M-SB mixed into 40 g of hydrogel CHO, which is a component of the “Bicarb System”. Repeated fingertip blood samples were collected at baseline and then every 30 min for 300 min. Each sample was obtained using heparin-coated glass clinitubes (70 μl) (Radiometer Medical Ltd., Denmark) and immediately analysed for blood pH, HCO_3_^−^, Na^+^, K^+^, Ca^2+^ and Cl^−^ using a blood gas analyser (ABL9, Radiometer Medical Ltd., Denmark). Participants were permitted to ingest water ad libitum and remained in a rested state throughout, with the amount recorded and replicated for each trial. At each 30 min timepoint gastrointestinal (GI) discomfort was also recorded for a range of symptoms using a visual analogue scale (VAS), as per previous research [18]. The GI symptoms assessed were nausea, flatulence, stomach cramping, belching, stomach ache, bowel urgency, diarrhoea, vomiting and stomach bloating, as well as an assessment of perceived thirst. Each VAS scale was scored between 0 and 10, with 0 representing no symptoms and 10 representing most severe symptoms. The ITTP was used due to previous research identifying this as a reliable measure of peak alkalosis [12, 21], and could enhance performance to a greater extent than a standardised time frame of ingestion [11, 22].

#### Supplement Ingestion

Prior to the first TT in each trial participants ingested either 0.3 g kg^−1^ BM M-SB or a placebo (PLA) containing 0.21 g kg^−1^ sodium chloride in vegetarian capsules (bulk, size 00, Bulk, Colchester, UK). In the PLA trial, the same hydrogel CHO product containing ~ 40 g of CHO was administered for blinding purposes (Maurten AB, Gothenburg, Sweden). For the timing of M-SB ingestion the ITTP HCO_3_^−^ was used and this timing was also used for the PLA trial (range 90–240 min). All participants completed ingestion within a 10-min window. The VAS scales for GI discomfort were taken at multiple time points, including baseline, ITTP, pre-TT1, recovery (15, 30 and 45 min), pre-TT2 and post-TT2. In the CON trial, participants did not ingest any SB or hydrogel CHO.

#### Time Trial Procedures

All cycling TTs were completed in a laboratory-controlled environment (ambient temperature ~ 18 °C ± 2 °C). Each trial consisted of completing 2 × 4 km cycling TTs interspersed with a 45-min passive recovery. The repeated 4 km TT protocol was selected as an appropriate exercise that significantly changes acid–base balance status [7, 9] and because the protocol can detect small changes in performance required for supplement studies (due to the high reliability of the protocol) [23]. Participants were permitted to select their preferred positions on the cycle ergometer (i.e. saddle and handlebar) and this was replicated for each TT. Each warm-up was individualised on the basis of the knowledge they were completing a 4 km TT, which was then recorded and replicated for each subsequent trial. Participants then completed each 4 km TT as quickly as possible and time elapsed was blinded. Only power output and cadence were visible throughout each TT. Whole body (RPE-O) and leg rating of perceived exertion (RPE-L) was recorded every 1 km of each TT (6–20 scale, [24]), along with heart rate (Polar, FT1, Finland). Throughout each trial, fingertip capillary blood samples were taken at baseline, ITTP, post-warm-up TT1 (PW1), post-TT1, during recovery (15-, 30- and 45-min recovery), post-warm-up TT2 (PW2) and post TT2. Time to complete, mean power output and mean speed were recorded for each TT.

Following each trial, a supplement belief questionnaire was administered as per previous research [18]. This questionnaire asked participants for a confidence score (0, no confidence; 5, not sure; 10, highest confidence) and which supplement they perceived they had ingested. Any score over 5 was considered a successful detection and this was then compared with the supplement they had truly ingested. In the CON trial, the supplement belief questionnaire was not administered.

### Statistical Analysis

Data were assessed for normality using Q–Q plots, histograms with normal distribution curves, and Shapiro–Wilk tests. Differences in mean values between conditions were assessed for TT performance (time, speed, power output and trial order), heart rate (HR) and all blood parameters using a series of repeated measure analysis of variance (ANOVA). Post hoc comparisons were made using a Bonferroni correction for multiple comparisons. Differences in TT performance, between TT1 and TT2 for each condition, were analysed using a comparison of the small main effects which provided additional post hoc pairwise comparisons. Ratings of perceived exertion were analysed using Friedman’s tests with Kendall’s *w* reported as an effect size. Total aggregated GI symptom scores are reported as arbitrary units (AU) and were analysed using a Wilcoxon test, with *r* reported as an effect size (where *r* = *z*/√*n*). Hedge’s *g*, *r* and *w* effect sizes were interpreted as small (0.2), medium (0.5) or large (0.8) and partial eta squared (*pη*^2^) effect sizes were interpreted as small (0.01), medium (0.06) or large (0.14) in accordance with Cohen [25]. All statistical procedures were performed using SPSS v29 for Windows (IBM, Chicago, USA), and statistical significance was assumed where *p* < 0.05.

## Results

### Time Trial Performance

**Fig. 3:**
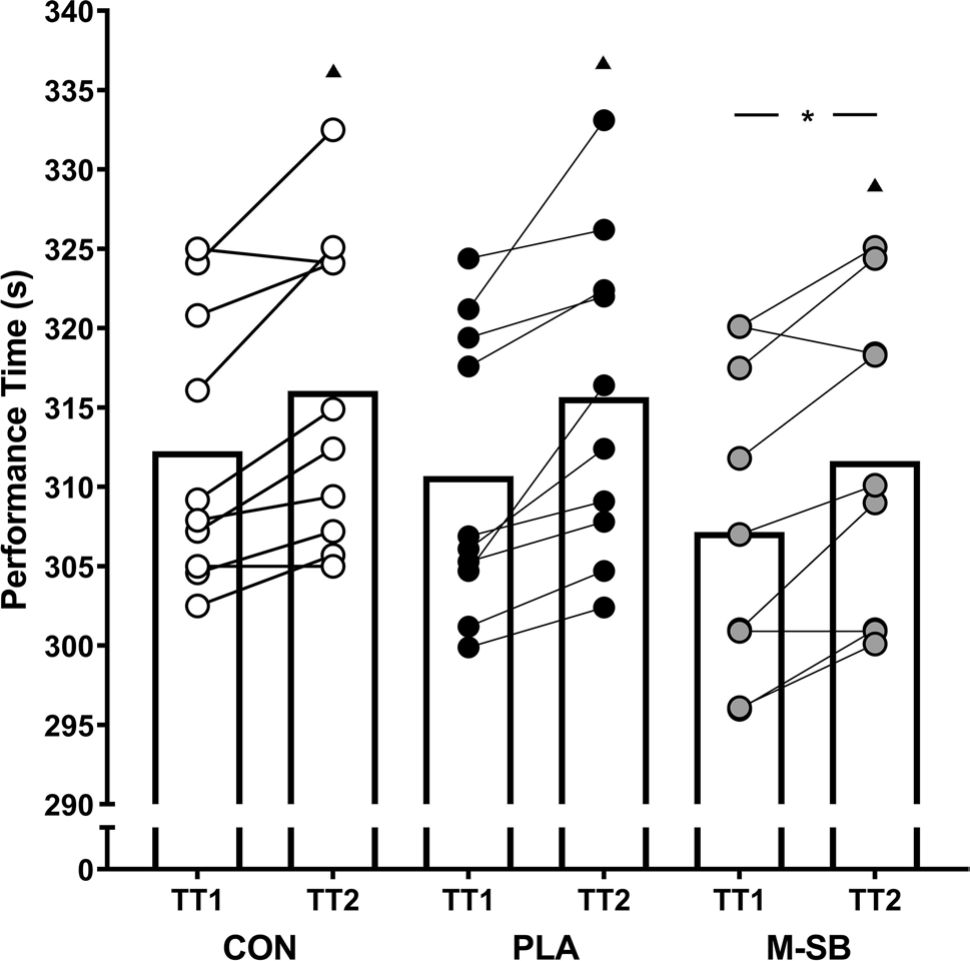
Mean and individual performance times in time trial (TT1) and time trial 2 (TT2) during the control (CON) placebo (PLA) and Maurten sodium bicarbonate (M-SB) experimental conditions. (Triangle) denotes a significant difference between TT1 and TT2; (asterisk) denotes a significantly faster performance compared with the other conditions

Performance times (Fig. 3) were significantly improved in the M-SB condition (*f* = 28.12, *p* < 0.001, *pη*^2^ = 0.76), with faster performance times compared with the CON (*p* < 0.001) and PLA (*p* = 0.003) conditions. Time trial 1 performance was faster in the M-SB condition (307.2 ± 9.1 s) compared with TT1 in CON (312.2 ± 8.1 s; *p* = 0.004) and PLA (310.7 ± 8.5 s; *p* < 0.001). In TT2, performance was also significantly faster in the M-SB condition (311.6 ± 9.0) compared with CON (316.0 ± 9.2 s; *p* = 0.018) or PLA (315.7 ± 9.6 s; *p* = 0.002). There was also a main effect for time, with performance significantly slower in TT2 compared with TT1 (*f* = 22.23, *p* = 0.001, *pη*^2^ = 0.71). Time trial performances were slower in TT2 compared with TT1 in CON (*p* < 0.001), PLA (*p* < 0.001) and M-SB (*p* < 0.001), but no interaction effect was observed (*f* = 0.71, *p* = 0.504, *pη*^2^ = 0.07). Time trial performance differences were also reflected in the speed (*f* = 15.51, *p* < 0.001, *pη*^2^ = 0.63) and power (*f* = 13.42, *p* < 0.001, *pη*^2^ = 0.60) data, with main effects for experimental condition (Table 1). This resulted in faster respective speeds and higher power output in the M-SB condition, compared with both CON (*p* = 0.002 and *p* = 0.003) and PLA (*p* = 0.030 and *p* = 0.026), but there were no differences in speed (*p* = 0.152) or power (*p* = 0.632) between CON and PLA. There was no observed trial order effect (*f* = 3.16, *p* = 0.067, *pη*^2^ = 0.26). The findings of the supplement belief questionnaire suggest that blinding was successful, as only two responses correctly identified the treatment, whilst most responses were ‘unsure’ (*n* = 14) or incorrect (*n* = 4).

**Table 1:** Mean (± SD) cycling performance parameters and end of time trial heart rate and perceived exertion responses in the three experimental conditions

Variable	Condition	TT1	TT2
Speed (km h^−1^)	CON	46.18 ± 1.17	45.77 ± 1.30^a^
	PLA	46.47 ± 1.29	45.80 ± 1.35^a^
	M-SB	46.84 ± 1.32^b^	46.25 ± 1.31^a.b^
Power output (W)	CON	366.6 ± 26.2	356.2 ± 27.4^a^
	PLA	370.7 ± 29.1	356.9 ± 28.8^a^
	M-SB	379.0 ± 29.1^b^	366.2 ± 28.9^a,b^
HR (b min^−1^)	CON	176 ± 10	178 ± 10
	PLA	180 ± 8	178 ± 8
	M-SB	180 ± 10	179 ± 9
RPE-O (AU)	CON	19.5 ± 1.3	19.6 ± 0.5
	PLA	19.8 ± 0.4	19.5 ± 0.7
	M-SB	19.5 ± 0.7	19.8 ± 0.4
RPE-L (AU)	CON	19.6 ± 1.3	19.8 ± 0.4
	PLA	19.8 ± 0.4	19.7 ± 0.5
	M-SB	19.5 ± 0.7	19.9 ± 0.3

CON, PLA and M-SB denote the control, placebo and Maurten sodium bicarbonate experimental conditions, respectively. TT1/TT2 denote the time trial number in the condition. (a) denotes a significant difference between TT1 and TT2; (b) denotes a significant difference in the M-SB condition

*HR* heart rate, *RPE-O* whole body rating of perceived exertion, *RPE-L*, leg rating of perceived exertion

### Heart Rate and Ratings of Perceived Exertion

There were no effects of the experimental conditions on the HR responses (Table 1) during the repeated 4 km TT protocol (*f* = 0.47, *p* = 0.631, *pη*^2^ = 0.05). This was characterised by no between-condition differences at the start of the TTs (*p* = 0.263) or the end (*p* = 1.00), but HR did change during the TTs (*f* = 109.05, *p* < 0.001, *pη*^2^ = 0.92) with significant elevations between the start and end of each TT (*p* < 0.001). The RPE responses were also unaffected by the experimental conditions (Table 1). The RPE-O responses were not different either at the end of TT1 (*χ*^2^ = 1.08, *p* = 0.584, *w* = 0.05) or TT2 (*χ*^2^ = 3.50, *p* = 0.174, *w* = 0.18). Similar responses were also observed for RPE-L at the end of TT1 and TT2 (*χ*^2^ = 2.92, *p* = 0.232, *w* = 0.15 and *χ*^2^ = 1.50, *p* = 0.472, *w* = 0.75, respectively).

### Blood Metabolite Responses

**Fig. 4:**
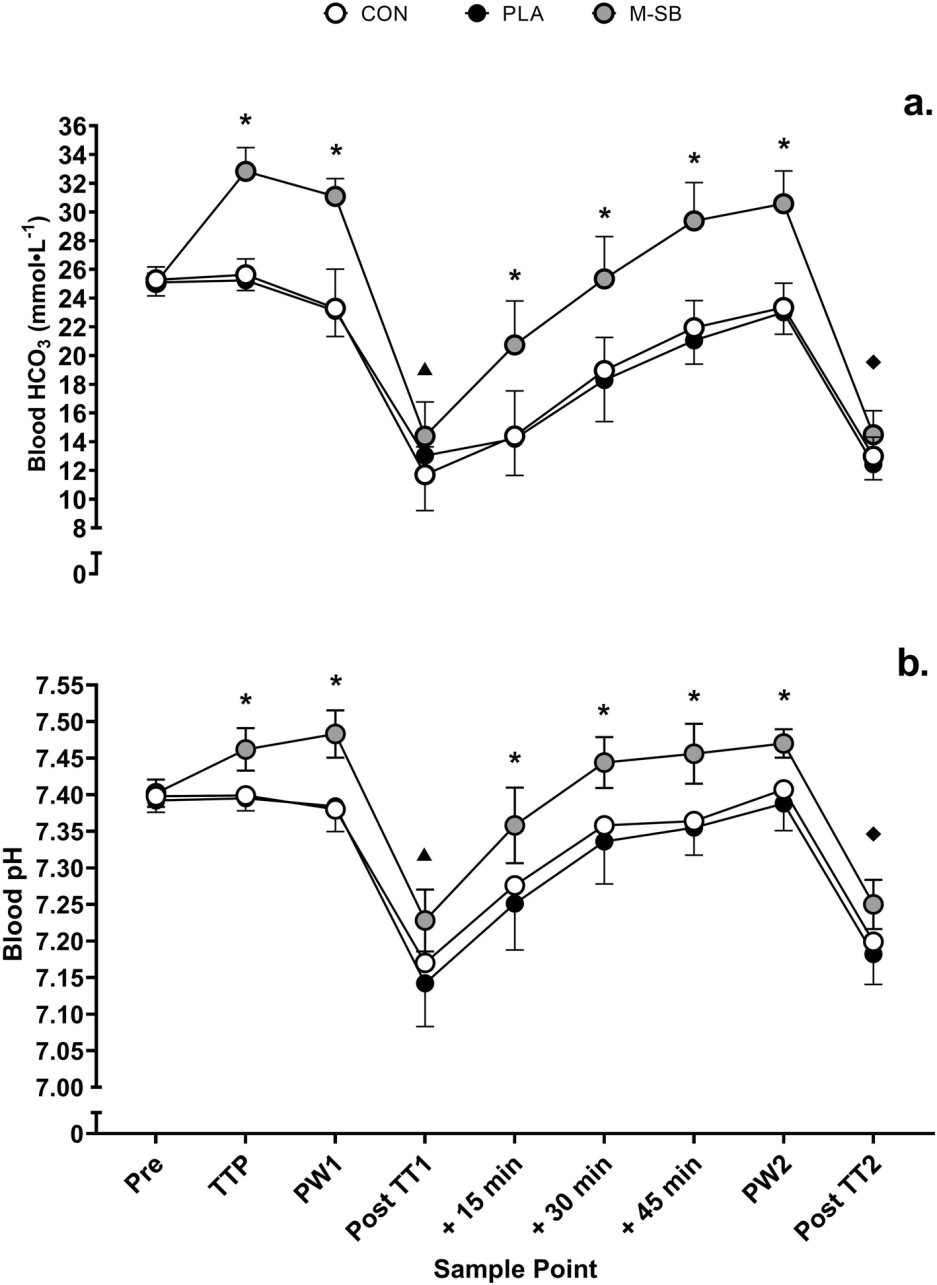
Mean (± SD) blood bicarbonate (HCO_3_) and pH responses to the two 4 km time trials (TT1 and TT2) during the control (CON) placebo (PLA) and Maurten sodium bicarbonate (M-SB) experimental conditions. (Asterisk) denotes a significant difference between M-SB and both CON and PLA conditions; (triangle) denotes a significant difference between M-SB and CON; (diamond) denotes a significant difference between M-SB and PLA. Pre, pre-exercise; TTP, time to peak; PW1, post-warm-up one; + 15 min, 15-min recovery; + 30 min, 30-min recovery; + 45 min, 45-min recovery; PW2, post-warm-up two

The mean absolute change in blood HCO_3_^−^ from baseline to ITTP was 8.0 ± 0.6 mmol L^−1^ in the ITTP trial and 7.7 ± 1.7 mmol L^−1^ at ITTP in the 2 × 4 km TT M-SB trial. This change in HCO_3_ concentration from baseline to ITTP was not different between actual ITTP and the suggested ITTP during the M-SB exercise trial (mean difference = 0.26 mmol L^−1^, *t* = 0.435, *p* = 0.067, *g* = 0.13) and appears reliable (ICC = 0.90). Time to peak HCO_3_^−^ was achieved in 129 ± 52 min, with a range of 90–240 min (median 105 min). Blood HCO_3_ responses (Fig. 4a) were significantly altered in response to the experimental conditions (*f* = 243.13, *p* < 0.001, *pη*^2^ = 0.96) and the TTs (*f* = 140.29, *p* < 0.001, *pη*^2^ = 0.94), and there was a significant condition × time interaction (*f* = 14.98, *p* < 0.001, *pη*^2^ = 0.63), with blood HCO_3_ elevated following M-SB ingestion (*p* < 0.01). In all trials HCO_3_ decreased considerably following each TT (*p* < 0.001), but its recovery was faster in the M-SB trial. Following TT1 increases in blood HCO_3_ were observed after 15 min (*p* < 0.001), but this was not the case both CON and PLA, which did not see significant changes to HCO_3_ in this initial recovery period (*p* = 0.491 and *p* = 1.00, respectively). The blood pH responses (Fig. 4b) were similar in nature to those observed for HCO_3_, where main effects were observed for condition (*f* = 128.66, *p* < 0.001, *pη*^2^ = 0.94) and time (*f* = 100.17, *p* < 0.001, *pη*^2^ = 0.92), and there was also condition × time interaction (*f* = 3.49, *p* < 0.001, *pη*^2^ = 0.28). This resulted in significantly elevated blood pH responses in M-SB compared with both CON (*p* < 0.001) and PLA (*p* < 0.001). There were no differences in the HCO_3_ (*p* = 0.938) or pH (*p* = 0.142) responses between the CON and PLA conditions.

**Fig. 5:**
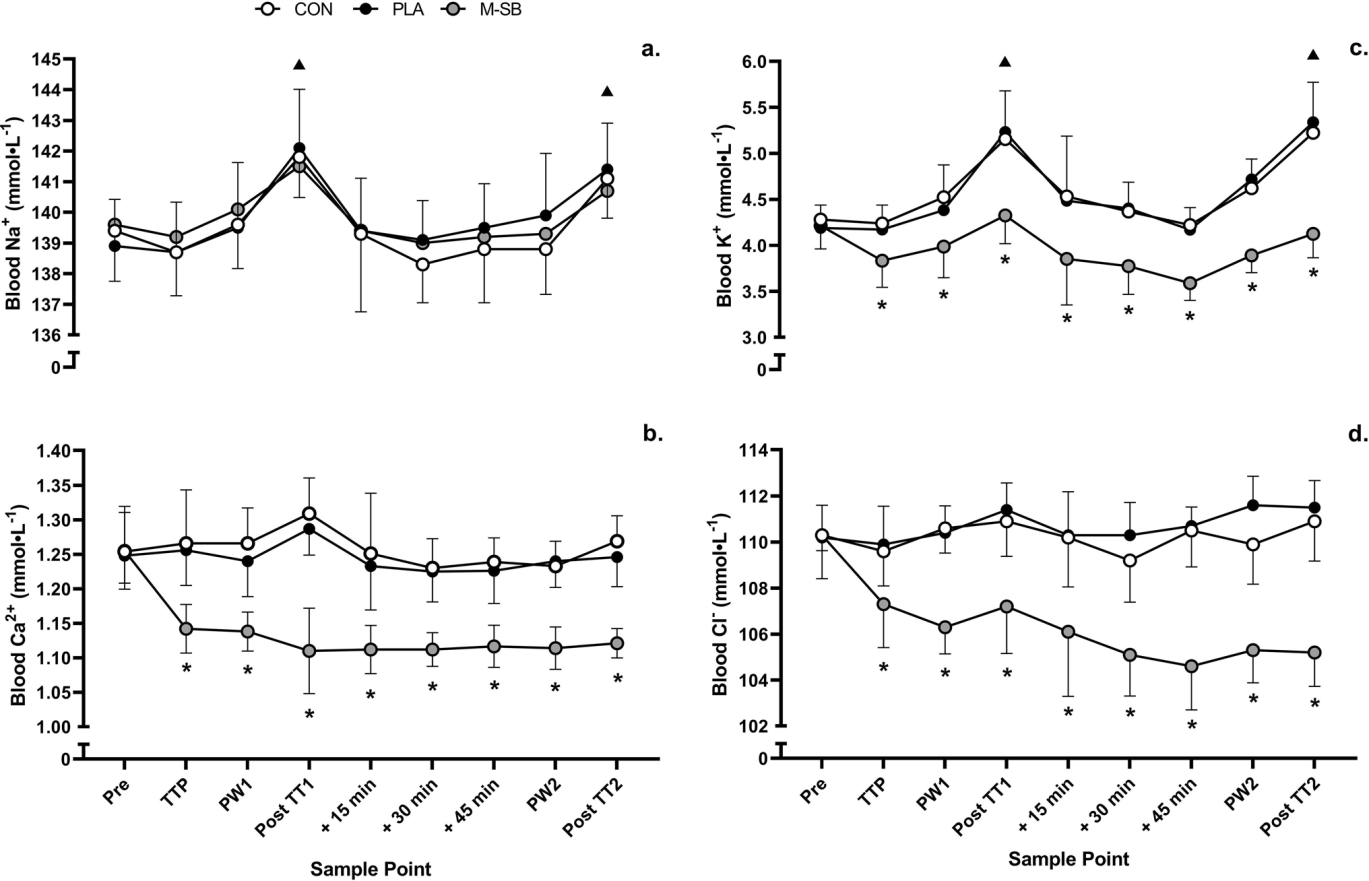
Mean (± SD) blood **a** sodium (Na^+^), **b** calcium (Ca^2+^), **c** potassium (K^+^), **d** chloride (Cl^−^) responses to the two 4 km time trials (TT1 and TT2) during the control (CON) placebo (PLA) and Maurten sodium bicarbonate (M-SB) experimental conditions. (Asterisk) denotes a significant difference between M-SB and both CON and PLA conditions; (triangle) denotes a significant increase from pre-exercise. *Pre* pre-exercise, *TTP* individual time to peak, *PW1* post-warm-up one; + 15 min, 15-min recovery; + 30 min, 30-min recovery; + 45 min, 45-min recovery, *PW2* post-warm-up two

The blood Na^+^ concentrations (Fig. 5a) were unaffected by the experimental conditions (*f* = 0.40, *p* = 0.679, *pη*^2^ = 0.04) but there was a main effect for time (*f* = 12.18, *p* < 0.001, *pη*^2^ = 0.58), caused by elevations in Na^+^ after TT1 (*p* = 0.015) and TT2 (*p* = 0.017). There was no condition × time interaction (*f* = 0.84, *p* = 0.641, *pη*^2^ = 0.09). Blood K^+^ (Fig. 5c) responded in a similar manner to that of Na^+^ to TT1 (*p* = 0.014) and TT2 (*p* = 0.037), resulting in a significant time effect (*f* = 17.16, *p* < 0.001, *pη*^2^ = 0.66). The K^+^, Ca^2+^ (Fig. 5b) and Cl^−^ (Fig. 5d) concentrations were also significantly lower from the ITTP sample point throughout the remainder of the trials in the M-SB condition (*f* = 128.36, *p* < 0.001, *pη*^2^ = 0.93; *f* = 123.96, *p* < 0.001, *pη*^2^ = 0.94; and *f* = 71.09, *p* < 0.001, *pη*^2^ = 0.89, respectively). The Ca^2+^ and Cl^−^ responses were not significantly altered in either the CON or PLA conditions. This altered electrolyte response also resulted in significant interaction effects (*f* = 4.34, *p* < 0.001, *pη*^2^ = 0.33; *f* = 7.73, *p* < 0.001, *pη*^2^ = 0.09; and* f* = 11.83, *p* < 0.001, *pη*^2^ = 0.57 for K^+^, Ca^2+^ and Cl^−^, respectively).

**Fig. 6:**
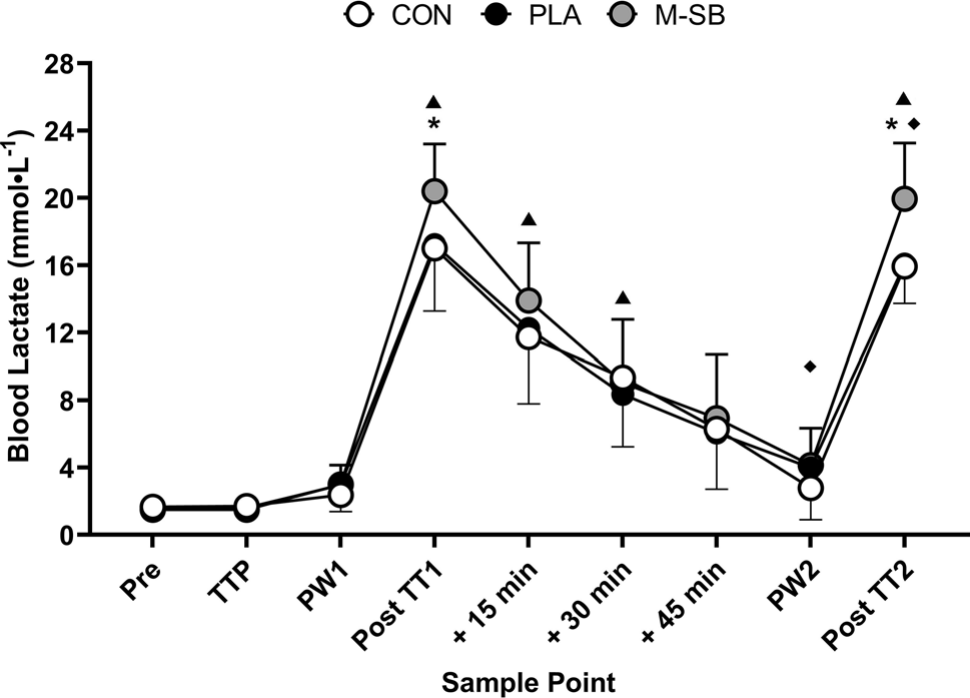
Mean (± SD) blood lactate responses to the two 4 km time trials (TT1 and TT2) during the control (CON) placebo (PLA) and Maurten sodium bicarbonate (M-SB) experimental conditions. (Asterisk) denotes a significant difference between PLA and M-SB; (diamond) denotes a significant difference between CON and M-SB; (triangle) denotes a significant change in concentration from the previous sample point. *Pre* pre-exercise, *TTP* individual time to peak, *PW1* post-warm-up one; + 15 min, 15-min recovery; + 30 min, 30-min recovery; + 45 min, 45-min recovery, *PW2* post-warm-up two

The blood lactate responses (Fig. 6) were significantly different between conditions (*f* = 3.97, *p* = 0.037, *pη*^2^ = 0.31), with higher lactate concentrations in the M-SB trials after TT1 (compared with the PLA; *p* = 0.037), at PW2 (compared with CON; *p* = 0.039) and again after TT2 (compared with CON and PLA, where *p* = 0.030 and *p* = 0.020, respectively). A main effect for time was also observed (*f* = 126.98, *p* < 0.001, *pη*^2^ = 0.93) with both TTs causing elevations in blood lactate concentration (*p* < 0.001). There were no differences in post-exercise blood lactate concentration between TT1 and TT2 (*p* = 1.000), although there was a significant condition × time interaction effect (*f* = 3.18, *p* < 0.001, *pη*^2^ = 0.26).

### Gastrointestinal and Perceived Thirst Responses

**Fig. 7:**
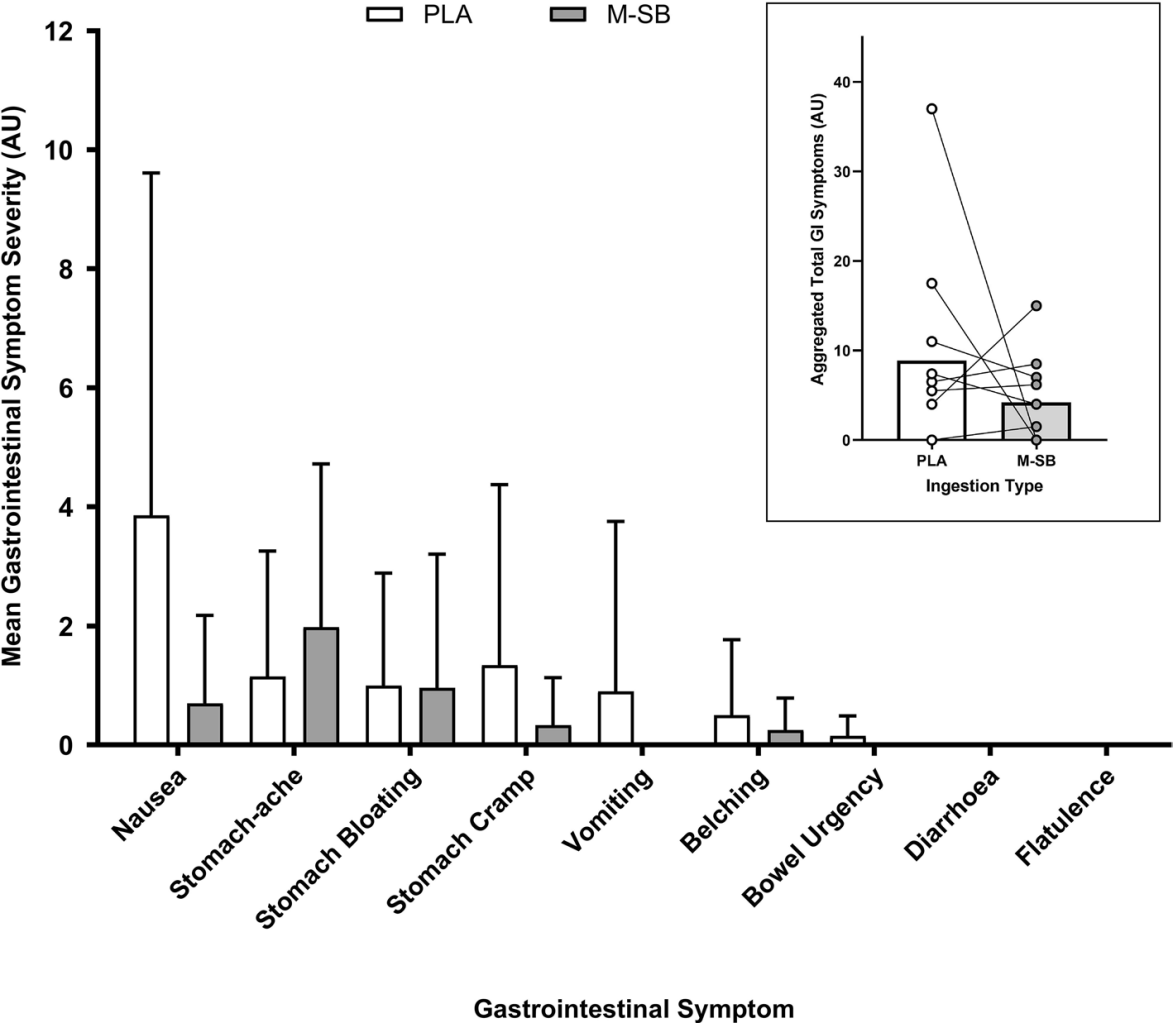
Mean (± SD) gastrointestinal (GI) symptom scores during the placebo (PLA) and Maurten sodium bicarbonate (M-SB) conditions. Inset: mean and individual total aggregated GI symptom responses to PLA and M-SB

**Fig. 8:**
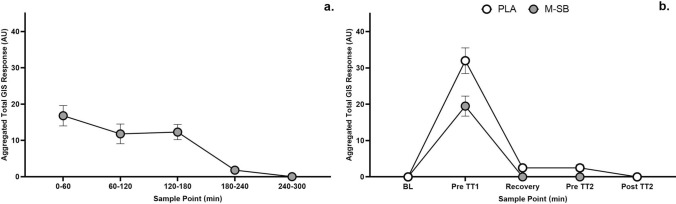
Aggregated total (± SD) gastrointestinal symptom (GIS) responses during the **a** ITTP trial (Maurten sodium bicarbonate (M-SB) only) and **b** exercise trials for the placebo (PLA) and Maurten sodium bicarbonate (M-SB) conditions. *BL* baseline, *TT1* time trial 1, *TT2* time trial 2, *Pre* pre-exercise, *TTP* individual time to peak, *PW1* post-warm-up one, + 15 min, 15-min recovery, + 30 min, 30-min recovery, + 45 min, 45-min recovery, *PW2* post-warm-up two

Following M-SB ingestion, the mean GI symptoms were elevated for nausea, stomach cramp and bloating, belching and stomach ache during the ITTP assessment (Fig. 7). The aggregated total GI symptoms response was more severe in the first hour of the TTP trial following M-SB ingestion, although was generally mild. During the exercise trials (Fig. 8), only mild GI symptoms were reported in both PLA and M-SB ingestion strategies, with the highest rating being 5/10 (stomach cramp) following PLA consumption and 4/10 (stomach cramp) after M-SB consumption. In the M-SB condition, total aggregated perceptions of thirst were slightly elevated (18 AU) compared with no elevations in thirst in the PLA condition (0 AU). Five participants reported elevated thirst following M-SB ingestion with the highest rating being 5/10. Elevated thirst was only present before TT1 and in the recovery period, but not before TT2 or after it.

## Discussion

The aim of this study was to investigate the effects of M-SB ingestion on repeated 4 km TT cycling performance in well-trained male cyclists. The findings of this study are the first to report that M-SB ingestion improves acid–base balance recovery and repeated cycling TT performance compared with a placebo. Compared with previous research using other forms of SB (e.g. solution, vegetarian capsules) acid–base balance recovery and exercise performance was improved to a similar extent in the current study [7–9, 16], whilst the HR and perceived exertion responses were unchanged by the different conditions. This suggests that the internal feedback used to regulate exercise intensity was the same, but M-SB ingestion allowed more effort to be invested for the same perceived exertion. Importantly, GI discomfort was also very low following M-SB ingestion, therefore combined with the performance enhancement, practitioners and athletes could consider using this product to induce performance benefits. This may be of even more relevance to those athletes with a history of GI discomfort following use of traditional methods, particularly if those side effects or those reported by others represent a barrier to ingesting SB.

The low GI discomfort ratings following M-SB ingestion, coupled with their complete absence for some participants within the cohort, is clearly an interesting finding. The most severe symptoms were nausea (mean ± SD: 0.8 ± 0.2 AU) and stomach ache (mean: 2.75 ± 0.25 AU) although these were only reported in a small number of participants (3/10). The current findings follow from previous research by Gough and Sparks [18] who reported GI discomfort was almost eliminated by M-SB ingestion compared with vegetarian capsule SB, albeit in a rested state. It would therefore appear that M-SB ingestion also results in low GI discomfort within an exercise context, which is a scenario where pre-exercise anxiety can correlate with exacerbated GI discomfort responses to exercise [26, 27]. These findings support the claims that this method of delivery reduces GI discomfort by allowing passage through the pyloric sphincter, thereby limiting the reaction of SB with stomach acid (due to the small diameter of the mini-tablet design and/or the hydrogel CHO) [18]. Future research should now assess the relationship between M-SB ingestion and GI discomfort in a variety of competitive scenarios in which higher natural levels of anxiety and arousal are present (compared with laboratory environments), which might increase GI responses. This is of particular importance given the anecdotal reports of widespread use of this product in cycling, swimming and athletics.

In line with previous research [18], M-SB ingestion increased pre-exercise blood HCO_3_^−^ above the 5 mmol L^−1^ increase purported to lead to ergogenic effects [15]. These changes in pH and HCO_3_^−^ prior to TT1 likely explain the ergogenic benefits observed in this study compared with PLA and CON. Furthermore, this study also reports that M-SB ingestion improves the post-exercise blood acid–base balance recovery. Notably, HCO_3_^−^ returned to pre-exercise concentrations following M-SB ingestion after 45 min of recovery, with most participants achieving their initial ITTP (~ 29 mmol L^−1^). In contrast, PLA and CON were below typical resting blood HCO_3_^−^ concentrations at that same timepoint (~ 21 and 22 mmol L^−1^). These changes are supported by the significant reduction in electrolytes Ca^2+^, Cl^−^ and K^+^ in the M-SB versus PLA and CON conditions. This evidence combined provides support for the likely ergogenic mechanism being present for both TT1 and TT2 and offer an explanation as to why performance was improved in both bouts. These findings agree with previous research [7, 9] that reported improved acid–base balance recovery and performance benefits following solution form SB, albeit in normobaric hypoxia (~ 3000 m). Interestingly, thirst perception was slightly elevated following M-SB, but these responses were also mild, and this is a typical observation following SB ingestion. Indeed, similar thirst responses have previously been reported using other SB ingestion types, and this is likely explained by the Na^+^ load that is present from both (0.21 g kg^−1^ BM Na^+^ per 0.3 g kg^−1^ BM dose of SB) [7, 18].

The findings of this study are comparable to previous research showing either single or repeated 4 km cycling TT performance was improved with SB ingestion when ingested at ITTP HCO_3_^−^ [7–9, 17, 17, 28]. Considering that studies using a standardised time of ingestion have reported no ergogenic effect of SB ingestion on 4 km cycling TT performance [29, 30], it is intuitive to suggest that individualising ingestion approaches may be optimal. Indeed, Boegman et al. [22] reported a small but significant improvement using an ITTP approach versus standardised timing in 2 km TT rowing, and Lopes-Silva [11] reported in a meta-analysis that mean power in cycling TTs was enhanced to a greater magnitude using ITTP versus standardised ingestion timing. However, not all participants benefited from the ITTP ingestion strategy, such that Boegman et al. [22] reported 7/23 did not gain any additional benefits over a standardised one. It is sensible to suggest that whilst the ITTP HCO_3_^−^ approach might increase the chance of securing an ergogenic benefit, the standardised approach is still a viable option for some. Of note, by 90 min post-ingestion in the current study, all participants had achieved a potentially ergogenic state of alkalosis (> 5 mmol L^−1^). Furthermore, the post-exercise recovery of blood HCO_3_^−^ concentrations suggests a robust ergogenic “window” is possible with M-SB ingestion. These two factors question the need to determine the ITTP when using M-SB, but at present this has not been directly established. Further research is therefore needed to compare a standardised versus an individualised ingestion timing of M-SB to assess whether the latter can provide greater magnitude of performance benefit.

The performance benefit reported in the current study does offer an ergogenic strategy to improve repeated high-intensity efforts, which is important due to the typical training and competition patterns of a cyclist. Specifically, cyclists may train twice per day with limited recovery, and therefore they could potentially use this strategy to improve performance. Equally, in competition there is usually limited time between either individual and team events, or heat and finals. For example, there were just 60 min separating the first round and the final at the 2016 Rio Olympics for the men’s 4 km team pursuit. It is important to note, however, that not all participants’ performance improved, as two were unchanged (< 0.9 s difference between M-SB and PLA). Interestingly, these two individuals were more endurance trained compared with the other cyclists in the sample (data not reported). Data in mice from Higgins et al. [31] reported the ergogenic benefits of SB ingestion were greater in extensor digitorum longus (type II) than soleus (type I). It is possible that muscle fibre type might explain why the two participants in the current did not improve their performance in either TT, however, more research is clearly required to investigate this further in humans.

This study offers valuable insight into the effects of M-SB ingestion on exercise performance, recovery and GI discomfort; however, the authors acknowledge the limitation that this supplement was not directly compared with other SB ingestion types. This would have added value to the comparisons with the previous literature that has used SB in solution or in a variety of capsule types. This was decided against due to the number of trials required, and including other forms of SB may have made the study susceptible to interference effects (such as training adaptations). Equally, a recent study has also compared the acid–base balance and GI discomfort responses between M-SB and capsule SB ingestion, although this was without a trial to investigate exercise performance responses [18]. It is also acknowledged that a sample size power calculation was not performed prior to the study. However, this was due to the lack of historical data on repeated 4 km TTs to determine the smallest effect size of interest, and the sample size was determined primarily on resource constraints [32].

## Conclusion

The primary novel findings of this study are that the ingestion of M-SB at ITTP HCO_3_^−^ improved repeated 4 km TT performance, with only very minor thirst and GI discomfort. It is likely that either the increased buffering capacity and/or the collective change in ionic movements explain the ergogenic benefits of M-SB. The ingestion of M-SB also increased the recovery of acid–base balance variables, including HCO_3_^−^, which is likely why subsequent performance was enhanced. Importantly, GI discomfort was nearly eliminated entirely following ingestion of M-SB and this is of clear practical benefit for practitioners and athletes. Collectively, ingestion of M-SB could be applied to both competition and/or training where only a short recovery is available such as during track cycling events, but further research is needed to evaluate this in other contexts.

## Declarations

### Availability of data and material

Data are available on request from the corresponding author.

## References

[1] McNaughton LR, Gough L, Deb S, Bentley D, Sparks SA. Recent developments in the use of sodium bicarbonate as an ergogenic aid. Curr Sports Med Rep. 2016;15(4):233–44. 27399820 10.1249/JSR.0000000000000283

[2] Bishop D, Edge J, Davis C, Goodman C. Induced metabolic alkalosis affects muscle metabolism and repeated-sprint ability. Med Sci Sports Exerc. 2004;36(5):807–13. 15126714 10.1249/01.mss.0000126392.20025.17

[3] Place N, Westerblad H. Metabolic factors in skeletal muscle fatigue. McConell. G. Exercise metabolism. Cham: Springer International Publishing; 2022. p. 377–99.

[4] Allen DG, Lamb GD, Westerblad H. Skeletal muscle fatigue: cellular mechanisms. Physiol Rev. 2008;88(1):287–332. 18195089 10.1152/physrev.00015.2007

[5] Allen DG, Lamb GD, Westerblad H. Impaired calcium release during fatigue. J Appl Physiol. 2008;104(1):296–305. 17962573 10.1152/japplphysiol.00908.2007

[6] Cairns SP, Lindinger MI. Do multiple ionic interactions contribute to skeletal muscle fatigue? J Physiol. 2008;586(17):4039–54. 18591187 10.1113/jphysiol.2008.155424 PMC2652190

[7] Gough LA, Brown D, Deb SK, Sparks SA, McNaughton LR. The influence of alkalosis on repeated high-intensity exercise performance and acid–base balance recovery in acute moderate hypoxic conditions. Eur J Appl Physiol. 2018;118:2489–98. 30196448 10.1007/s00421-018-3975-z PMC6244684

[8] Gough LA, Deb SK, Sparks SA, McNaughton LR. Sodium bicarbonate improves 4 km time trial cycling performance when individualised to time to peak blood bicarbonate in trained male cyclists. J Sports Sci. 2018;36(15):1705–12. 29183257 10.1080/02640414.2017.1410875

[9] Gough LA, Deb SK, Brown D, Sparks SA, McNaughton LR. The effects of sodium bicarbonate ingestion on cycling performance and acid base balance recovery in acute normobaric hypoxia. J Sports Sci. 2019;37(13):1464–71. 30668281 10.1080/02640414.2019.1568173

[10] Siegler JC, Hirscher K. Sodium bicarbonate ingestion and boxing performance. J Strength Cond Res. 2010;24(1):103–8. 19625976 10.1519/JSC.0b013e3181a392b2

[11] Lopes-Silva JP, Correia-Oliveira CR. Acute effects of sodium bicarbonate ingestion on cycling time-trial performance: a systematic review and meta-analysis of randomized controlled trials. Eur J Sport Sci. 2023;23(6):943–54. 35633035 10.1080/17461391.2022.2071171

[12] Gough LA, Deb SK, Sparks AS, McNaughton LR. The reproducibility of blood acid base responses in male collegiate athletes following individualised doses of sodium bicarbonate: a randomised controlled crossover study. Sports Med. 2017;47:2117–27. 28229390 10.1007/s40279-017-0699-x

[13] Saunders B, Sale C, Harris RC, Sunderland C. Sodium bicarbonate and high-intensity-cycling capacity: variability in responses. Int J Sports Physiol Perform. 2014;9(4):627–32. 24155093 10.1123/ijspp.2013-0295

[14] Deb SK, Gough LA, Sparks SA, McNaughton LR. Sodium bicarbonate supplementation improves severe-intensity intermittent exercise under moderate acute hypoxic conditions. Eur J Appl Physiol. 2018;118:607–15. 29344729 10.1007/s00421-018-3801-7 PMC5805802

[15] Carr AJ, Slater GJ, Gore CJ, Dawson B, Burke LM. Effect of sodium bicarbonate on [HCO3−], pH, and gastrointestinal symptoms. Int J Sport Nutr Exerc Metab. 2011;21(3):189–94. 21719899 10.1123/ijsnem.21.3.189

[16] Hilton NP, Leach NK, Sparks SA, Gough LA, Craig MM, Deb SK, McNaughton LR. A novel ingestion strategy for sodium bicarbonate supplementation in a delayed-release form: a randomised crossover study in trained males. Sports Med Open. 2019;5(1):1–8. 30680463 10.1186/s40798-019-0177-0 PMC6346694

[17] Hilton NP, Leach NK, Hilton MM, Sparks SA, McNaughton LR. Enteric-coated sodium bicarbonate supplementation improves high-intensity cycling performance in trained cyclists. Eur J Appl Physiol. 2020;120:1563–73. 32388584 10.1007/s00421-020-04387-5 PMC7295736

[18] Gough LA, Sparks SA. The effects of a carbohydrate hydrogel system for the delivery of bicarbonate mini-tablets on acid–base buffering and gastrointestinal symptoms in resting well-trained male cyclists. Sports Med Open. 2024;10(1):1–9. 38356036 10.1186/s40798-024-00684-x PMC10866843

[19] De Pauw K, Roelands B, Cheung SS, De Geus B, Rietjens G, Meeusen R. Guidelines to classify subject groups in sport-science research. Int J Sports Physiol Perform. 2013;8(2):111–22. 23428482 10.1123/ijspp.8.2.111

[20] Costello N, Deighton K, Dyson J, Mckenna J, Jones B. Snap-N-Send: a valid and reliable method for assessing the energy intake of elite adolescent athletes. Eur J Sport Sci. 2017;17(8):1044–55. 28627289 10.1080/17461391.2017.1337815

[21] Newbury JW, Cole M, Kelly AL, Chessor RJ, Sparks SA, McNaughton LR, Gough LA. The time to peak blood bicarbonate (HCO3–), pH, and the strong ion difference (SID) following sodium bicarbonate (NaHCO_3_) ingestion in highly trained adolescent swimmers. PLoS One. 2021;16(7): e0248456. 34197456 10.1371/journal.pone.0248456 PMC8248647

[22] Boegman S, Stellingwerff T, Shaw G, Clarke N, Graham K, Cross R, Siegler JC. The impact of individualizing sodium bicarbonate supplementation strategies on world-class rowing performance. Front Nutr. 2020;7:138. 33015117 10.3389/fnut.2020.00138 PMC7509055

[23] Stone MR, Thomas K, Wilkinson M, St. Glair Gibson A, Thompson KG. Consistency of perceptual and metabolic responses to a laboratory-based simulated 4,000-m cycling time trial. Eur J Appl Physiol. 2011;111:1807–13. 21222130 10.1007/s00421-010-1818-7

[24] Borg G. Ratings of perceived exertion and heart rates during short-term cycle exercise and their use in a new cycling strength test. Int J Sports Med. 1982;3(03):153–8. 7129724 10.1055/s-2008-1026080

[25] Cohen J. Statistical power analysis for the behavioural sciences. 2nd ed. Hillsdale: Lawrence Erlbaum Associates; 1988. p. 567.

[26] Wilson PB, Russell H, Pugh J. Anxiety may be a risk factor for experiencing gastrointestinal symptoms during endurance races: an observational study. Eur J Sport Sci. 2021;21(3):421–7. 32251613 10.1080/17461391.2020.1746836

[27] Wynne JL, Wilson PB. Thorn in your side or thorn in your head? Anxiety and stress as correlates of exercise-related transient abdominal pain. Clin J Sport Med. 2022;32(5):471–5. 36083326 10.1097/JSM.0000000000001000

[28] Gough LA, Williams JJ, Newbury JW, Gurton WH. The effects of sodium bicarbonate supplementation at individual time-to-peak blood bicarbonate on 4-km cycling time trial performance in the heat. Eur J Sport Sci. 2022;22(12):1856–64. 34704539 10.1080/17461391.2021.1998644

[29] Callahan MJ, Parr EB, Hawley JA, Burke LM. Single and combined effects of beetroot crystals and sodium bicarbonate on 4-km cycling time trial performance. Int J Sport Nutr Exerc Metab. 2017;27(3):271–8. 27834492 10.1123/ijsnem.2016-0228

[30] Correia-Oliveira CR, Lopes-Silva JP, Bertuzzi R, Mcconell GK, Bishop DJ, Lima-Silva AE, Kiss MAPDM. Acidosis, but not alkalosis, affects anaerobic metabolism and performance in a 4-km time trial. Med Sci Sports Exerc. 2017;49(9):1899–910. 28398947 10.1249/MSS.0000000000001295

[31] Higgins MF, James RS, Price MJ. The effects of sodium bicarbonate (NaHCO_3_) ingestion on high intensity cycling capacity. J Sports Sci. 2013;31(9):972–81. 23323673 10.1080/02640414.2012.758868

[32] Lakens D. Sample size justification. Collabra Psychol. 2022;8(1):33267.

